# Topical Biological Agents as Adjuncts to Improve Wound Healing in Chronic Diabetic Wounds: A Systematic Review of Clinical Evidence and Future Directions

**DOI:** 10.7759/cureus.27180

**Published:** 2022-07-23

**Authors:** Andrew Yew Wei Wong, Bernard Soon Yang Ong, Ainsley Ryan Yan Bin Lee, Aaron Shengting Mai, Sathiyamoorthy Selvarajan, Satish R Lakshminarasappa, Sook Muay Tay

**Affiliations:** 1 Yong Loo Lin School of Medicine, National University of Singapore, Singapore, SGP; 2 Anatomical Pathology, Singapore General Hospital, Singapore, SGP; 3 Anatomy, National University of Singapore, Singapore, SGP; 4 Surgical Intensive Care, Singapore General Hospital, Singapore, SGP

**Keywords:** wound healing, human umbilical cord, platelet-rich fibrin, vascular endothelial growth factor, epidermal growth factor, fibroblast growth factor, platelet-derived growth factor, diabetic foot ulcer

## Abstract

Diabetes is a leading chronic illness in the modern world and 19-34% develop chronic diabetic foot ulcers (DFUs) in their lifetime, often necessitating amputation. The reduction in tissue growth factors and resulting imbalance between proteolytic enzymes and their inhibitors, along with systemic factors impairing healing appear particularly important in chronic wounds. Growth factors applied topically have thus been suggested to be a non-invasive, safe, and cost-effective adjunct to improve wound healing and prevent complications.

Comprehensive database searches of MEDLINE via PubMed, EMBASE, Cochrane, and ClinicalTrials.gov were performed to identify clinical evidence and ongoing trials. The risk of bias analysis included randomized controlled trials (RCTs) was performed using the Cochrane Risk of Bias 2.0 tool. We included randomized controlled trials that compared the use of a topical biologic growth factor-containing regimen to any other regimen. Primary outcomes of interest were time to wound closure, healing rate, and time. Secondary outcomes included the incidence of adverse events such as infection.

A total of 41 trials from 1992-2020 were included in this review, with a total recorded 3,112 patients. Platelet-derived growth factors (PDGF) in the form of becaplermin gel are likely to reduce the time of closure, increase the incidence of wound closure, and complete wound healing. Human umbilical cord-related treatments, dehydrated human amnion and chorion allograft (dHACA), and hypothermically stored amniotic membrane (HSAM), consistently increased the rates and incidence of complete ulcer healing while reducing ulcer size and time to complete ulcer healing. Fibroblast growth factor-1 (FGF1) showed only a slight benefit in multiple studies regarding increasing complete ulcer healing rates and incidence while reducing ulcer size and time to complete ulcer healing, with a few studies showing no statistical difference from placebo. Platelet-rich fibrin (PRF) is consistent in reducing the time to complete ulcer healing and increasing wound healing rate but may not reduce ulcer size or increase the incidence of complete ulcer healing.

Targeting the wound healing pathway via the extrinsic administration of growth factors is a promising option to augment wound healing in diabetic patients. Growth factors have also shown promise in specific subgroups of patients who are at risk of significantly impaired wound healing such as those with a history of secondary infection and vasculopathy. As diabetes impairs multiple stages of wound healing, combining growth factors in diabetic wound care may prove to be an area of interest. Evidence from this systematic literature review suggests that topical adjuncts probably reduce time to wound closure, reduce healing time, and increase the healing rate in patients with chronic DFUs.

## Introduction and background

Disease burden and significance

Diabetes mellitus is an important chronic illness that affects 422 million people in the world [1]. It is well-studied that diabetes mellitus increases the risk of multiple complications [2], a significant complication being the development of a diabetic foot ulcer (DFU). A DFU is a common, highly morbid, and costly condition. It is estimated that 25% of the diabetic population will develop DFU complications in their lifetime [3]. It is one of the leading causes of lower extremity amputations, with more than 50% of diabetic ulcers becoming infected, of which 20% result in amputations [4]. Patients with DFUs suffer a significant drop in quality of life [5] and are more likely to experience monetary losses [6].

Wound healing and pathophysiology of diabetic foot ulcers

A wound is defined as a disruption of the normal structure and function of the skin with its adjacent underlying soft tissue structures. Acute wounds typically heal in a structured and effective manner classified by overlapping phases of inflammation, epithelialization, fibroplasia, and maturation. This involves angiogenesis from structured cell migration and the recruitment of endothelial cells. There are many growth factors and cytokines released by these cell types that coordinate and maintain wound healing [7].

The focus of this paper is on chronic wounds, as they affect a substantial proportion of the population and contribute to a significant economic burden [6]. These wounds usually have chronic inflammation and fail to heal. The normal physiology is transformed into the pathophysiology of a chronic cycle, without a distinct wound closure endpoint. In diabetic patients, this is further complicated by peripheral artery disease (PAD), which can cause motor and sensory neuropathy. Motor neuropathy may result in skin erosion and ulceration [8]. Sensory neuropathy may result in patients being unaware of sustained injuries due to the loss of nociception and delayed health-seeking behavior [9].

The risk factors of PAD in diabetic patients reduce the likelihood of wounds receiving adequate blood supply necessary to achieve proper wound healing. It is well-studied that sufficient blood supply is essential for wound healing [10]. Blood provides the components required for structured cell migration, recruitment of endothelial cells, and growth factor and cytokine release. In diabetes, insulin resistance, hyperinflammatory and hyperglycemic states contribute to increased inflammation [11-12], endothelial dysfunction [13-16], enhanced vasoconstriction [17], enhanced thrombosis [18-19], and impaired growth factor production [20]. These complications impede blood supply and cause chronic wounds.

In this paper, we will focus on the topic of impaired growth factor production [20]. The reduction in tissue growth factors appears particularly important in DFUs and may partially explain why some wounds fail to heal. Chronic ulcers have been described to have reduced levels of platelet-derived growth factor (PDGF), basic fibroblast growth factor (bFGF), epidermal growth factor, and transforming growth factor β compared to acute wounds. It has been suggested that growth factors may become trapped by extracellular matrix molecules or may be degraded by proteases to an excessive degree, resulting in non-healing [21].

Background of intervention

This review seeks to elucidate some of the more promising and effective biologics that decrease the time to wound healing and percentage of wound recovery. The current standard practices in diabetic foot ulcer management include surgical debridement, facilitation of moist wound environment through dressings, exudate control, wound off-loading, vascular assessment, and improvement of glycemic control [22-23]. Despite these methods of wound control, there is still room for improvement in the outcomes of diabetic foot ulcers, considering that 20% of patients have unhealed diabetic foot ulcers within a year [24]. In this review, we report the recent advancements in biologics and emerging clinical trials regarding topical biologics for wound healing.

Platelet-derived growth factors/platelet-derived wound healing formula

In vitro, PDGF stimulates chemotaxis, proliferation, and novel gene expression in monocytes-macrophages and fibroblasts, cell types considered essential for tissue repair [25]. The biological effects of PDGF are exerted by activating two tyrosine kinase receptors [26]. Platelet-derived growth factor-BB (PDGF-BB) is considered to be the most effective isoform for wound healing [27]. Multiple studies have reported accelerating healing and increasing wound closure in diabetic and non-diabetic wound models with PDGF administration. Various biomaterials and delivery systems are capable of administering PDGF.

Human umbilical cord/amniotic membrane

Four components of the human umbilical cord (HUC) have been identified. The amniotic epithelial membrane (AM), the sub-amnion or “cord lining” (SA), Wharton's Jelly (WJ), and the perivascular region (PV) surrounding the umbilical blood vessels [28]. Each compartment has been described to contain mesenchymal stem cells (MSCs) with different characteristics. However, the role of MSCs in HUC has not been fully explained; furthermore, isolating MSCs from each compartment is difficult. The human umbilical cord contains factors that stimulate cell proliferation, migration, tissue differentiation, and growth.

The first reported use of HUC was as a patch to successfully treat gastroschisis in pediatric surgery [28]. Since then, it has become a tissue of great and increasing interest in regenerative medicine. HUC byproducts, such as cells and extracts, have been studied in vitro and in vivo with optimistic tissue results. HUC MSC, especially those isolated from WJ, are currently used in clinical trials that have reported safety and efficacy in wound healing [29].

Human basic fibroblast growth factor

The fibroblast growth factor (FGF) family is produced by keratinocytes, fibroblasts, endothelial cells, smooth muscle cells, chondrocytes, and mast cells. FGF-2 also known as basic fibroblast growth factor (bFGF) is increased in acute wounds and plays a role in granulation tissue formation, re-epithelialization, and tissue remodeling [30].

Acidic FGF (aFGF) and bFGF were originally purified from the brain and pituitary gland as growth factors for fibroblasts [31-32]. Of particular interest, the topical application of recombinant human bFGF (rh-bFGF) has shown promise in the management of DFUs as well as second-degree burns. There are currently, however, few studies examining the efficacy of rh-FGF in the treatment of ischemic DFUs. Studies in China often use recombinant bovine bFGF (rb-bFGF).

Recombinant human vascular endothelial growth factor

Vascular endothelial growth factor (VEGF) is a homodimeric glycoprotein that shares almost 20% amino acid homology with PDGF. VEGF’s role in wound healing includes stimulation of angiogenesis [33]. VEGF plays a role in several of these processes within angiogenesis, including functioning as an endothelial cell mitogen, a chemotactic agent, and an inducer of vascular permeability. The clinical significance of adequate VEGF production during wound repair has been repeatedly demonstrated [34-35].

Initially, a balloon transfer of plasmid DNA expressing VEGF165 was attempted on a non-diabetic patient with the arterial occlusive disease in the lower extremity [33]. VEGF since has shown effective results administered alone or as adjunctive therapy to angioplasty and surgery. VEGF has shown promise in non-healing skin ulcers, with its effects on multiple components of the wound healing cascade.

Platelet-rich fibrin

Platelet-rich fibrin (PRF) is useful in wound healing and skin rejuvenation as primary and supplemental techniques. With diverse, and increasingly pertinent, capacities in aesthetic medicine and surgery, PRF is simple to obtain, inexpensive, and may be administered topically, injected, or in conjunction with other aesthetic procedures [36]. PRF allows the prolonged release of growth factors attributed to its fibrin matrix, cellular components, and prolonged release of growth factors. Without anticoagulants, PRF spontaneously forms a fibrin matrix gelatinous clot that confines growth factor secretion to the clotting site. In tissue repair, recruited fibroblasts initiate collagen synthesis and reorganize the fibrin matrix. Thus, the combined effects of growth factor secretion and fibroblast recruitment in PRF work synergistically to promote collagenesis and tissue regeneration. Because the fibrin matrix is better organized, it can more efficiently direct stem cell migration and the healing program [37].

PRF serves as a supportive template for tissue regeneration by guiding clot formation through sustaining growth factors and stem cells as a naturally forming fibrin scaffold. There are many applications of PRF in cosmetic medicine and surgery [38-39]. Further research is expected to uncover more benefits to be obtained from PRF’s regenerative properties, bioavailability, and autologous nature.

Epidermal growth factor

Epidermal growth factor (EGF) is a signaling protein that stimulates cell growth and differentiation by binding to its receptor. EGF participates in dermal wound healing through stimulation, proliferation, and migration of keratinocytes, endothelial cells, and fibroblasts and facilitates dermal regeneration [40].

It was first discovered during studies of nerve growth factors as a side effect of other experiments [41]. Studies related to EGF and its signaling pathway have extended to a broad range of investigations concerning its biological and pathophysiological roles in the development and human diseases, with further progression into clinical practice in the treatment of wounds. As EGF is readily degraded in the chronic wound environment, the development of EGF in wound healing has progressed toward the treatment of acute wounds. However, there is a recent focus of research on novel drug delivery systems capable of protecting and stabilizing the protein. The potential healing effects of EGF are at the forefront of research [42].

## Review

Methods

Search Strategy

We searched for articles in three electronic databases, PubMed, EMBASE, and Cochrane Library, from inception to October 2021. The full search strategy is detailed in Table 1.

**Table 1:** Full search strategy Articles were searched from PubMed, Embase, and Cochrane Library, from inception to October 2021.

Database	Search term	No. of results
PubMed	(diabetes[title/abstract] or dm[title/abstract] or mellitus[title/abstract] or diabetic[title/abstract] or t2dm[title/abstract] or type 2[title/abstract] or type ii[title/abstract]) AND (topical[title/abstract] or platelet-derived[title/abstract] or pdgf[title/abstract] or rhpdgf[title/abstract] or pdgf*[title/abstract] or platelet[title/abstract] or becaplermin[title/abstract] or regranex[title/abstract] or plermin[title/abstract] or salidroside[title/abstract] or ttax*[title/abstract] or ttax01[title/abstract] or crypreserved[title/abstract] or umbilical cord[title/abstract] or amniotic[title/abstract] or fgf[title/abstract] or fibroblast[title/abstract] or growth factor[title/abstract] or fiblast[title/abstract] or trafermin[title/abstract] or fhbfgf[title/abstract] or recombinant[title/abstract] or trafermin[title/abstract] or telbermin[title/abstract] or fibrin[title/abstract] or prf[title/abstract] or vivostat[title/abstract] or growth factor[title/abstract] or vegf[title/abstract] or vascular endothelial[title/abstract]) AND (wound or ulcer or epithelial defect or injury or lesion) AND (random* or trial or control*)	3653
EMBASE	(diabetes:ti,ab or dm:ti,ab or mellitus:ti,ab or diabetic:ti,ab or t2dm:ti,ab or type 2:ti,ab or type ii:ti,ab) AND (topical:ti,ab or platelet-derived:ti,ab or pdgf:ti,ab or rhpdgf:ti,ab or pdgf*:ti,ab or platelet:ti,ab or becaplermin:ti,ab or regranex:ti,ab or plermin:ti,ab or salidroside:ti,ab or ttax*:ti,ab or ttax01:ti,ab or crypreserved:ti,ab or umbilical cord:ti,ab or amniotic:ti,ab or fgf:ti,ab or fibroblast:ti,ab or growth factor:ti,ab or fiblast:ti,ab or trafermin:ti,ab or fhbfgf:ti,ab or recombinant:ti,ab or trafermin:ti,ab or telbermin:ti,ab or fibrin:ti,ab or prf:ti,ab or vivostat:ti,ab or growth factor:ti,ab or vegf:ti,ab or vascular endothelial:ti,ab) AND (wound or ulcer or epithelial defect or injury or lesion) AND (random* or trial or control*) NOT medline/lim	3435
CENTRAL	(diabetes or dm or mellitus or diabetic or t2dm or type 2 or type ii) in Title Abstract Keyword AND (topical or platelet-derived or pdgf or rhpdgf or pdgf* or platelet or becaplermin or regranex or plermin or salidroside or ttax* or ttax01 or crypreserved or umbilical cord or amniotic or fgf or fibroblast or growth factor or fiblast or trafermin or fhbfgf or recombinant or trafermin or telbermin or fibrin or prf or vivostat or growth factor or vegf or vascular endothelial) in Title Abstract Keyword AND (wound or ulcer or epithelial defect or injury or lesion) in All Text AND (random* or trial or control*) in All Text	4135

Study Selection

**Figure 1:**
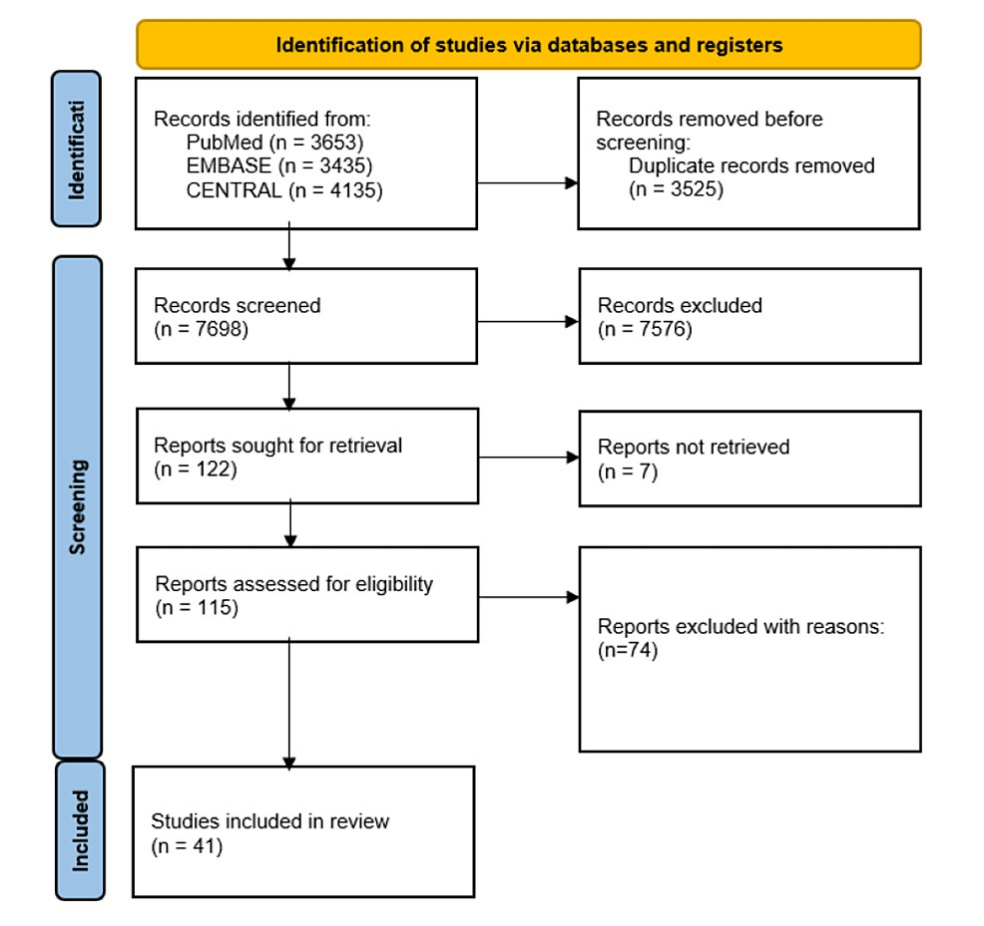
PRISMA flowchart PRISMA: Preferred Reporting Items for Systematic Reviews and Meta-Analyses

Four reviewers independently screened titles and abstracts to determine whether they met the eligibility criteria, with discrepancies resolved by consultation of a fifth reviewer. The reviewers then screened through the full texts and narrowed down the papers again for subsequent data extraction. The PRISMA flow diagram was used to summarise the study selection process in Figure 1.

Exclusion criteria: Studies were excluded for the following reasons: Not a randomized controlled trial, the paper did not involve information on human subjects, the trial has not been published in any peer-reviewed journal, the paper was published only in gray literature, the trial did not measure or report any of the aforementioned intervention-related outcomes.

Inclusion criteria: A randomized controlled trial (RCT) of any phase, published in a peer-reviewed journal that has been indexed by at least one of the databases searched, the trial uses a topic biologic agent as part of any arm of the intervention, the full manuscript is available in English, patients were included based on the treatment of peripheral wounds and have type I or II diabetes mellitus, the trial should have measured and reported at least one of the following intervention-related outcomes: (a) complete healing of the foot ulcer; (b) time to complete healing of the diabetic foot ulcer; (c) patient-reported mobility; (d) ulcer durability; (e) wound infection; (f) patient-reported quality of life/pain.

Data Extraction

Two reviewers used a predefined data extraction sheet to independently extract data on trial characteristics, population baseline characteristics, and outcomes from each included study. Trial characteristics included author, publication year, number of people in the control and intervention groups, drug used, frequency of dosage, and length of intervention. Baseline characteristics included subject ages and sex.

Risk of Bias Assessment

The Jadad score was used to assess the quality of included studies and consists of three items: randomization (0-2 points), blinding (0-2 points), and participant dropout and withdrawal (0-1 points). The final score ranges from 0 to 5 points with higher scores indicating better quality. Studies rated to have a score of 2 or less were considered to be of low quality and those with a score of 3 or more were considered to be of high quality [43].

Two reviewers assessed each included study independently. All discrepancies were resolved by involving a third reviewer to assess the study independently. The studies are detailed in Table 2.

**Table 2:** Quality and Risk-of-Bias Assessment According to the Jadad Scale

	Randomization			Blinding				
Study	Mentioned	Appropriate	Deduct 1 point if randomization is inappropriate	Mentioned	Appropriate	Deduct 1 point if blinding is inappropriate	Number of and reasons for withdrawal	Overall (/5)
PDGF								
Wieman et al (1998) [44]	1	1		1	1		1	5
Steed et al (2006) [45]	1			1				2
Niezgoda et al (2005) [46]	1	1						2
Landsman (2010) [47]	1	1		1		1		2
Ma et al (2015) [48]	1			1				2
Melba S et al (2016) [49]	1	1						2
HUC/TTAX01								
Glat P et al (2019) [50]	1	1		1	1		1	5
Serena TE et al (2019) [51]	1	1					1	3
Zelen, C. M. et al (2015) [52]	1	1		1	1		1	5
DiDomenico LA et al (2018) [53]	1	1		1	1		1	5
Tettelbach, W. et al (2019) [54]	1	1		1	1		1	5
Tettelbach, W. et al (2019) [55]	1	1		1	1		1	5
Snyder, R. J. et al (2016) [56]	1	1					1	3
Human basic fibroblast growth factor								
Uchi et al (2009) [57]								
Morimoto N et al (2013) [58]	1	1		1	1		1	5
Steed DL et al (1992)[59]				1				1
Santoro et al (2018) [60]	1	1						2
Olympus Biotech Corporation (Trafermin North) (2010) [61]	1	1		1	1		1	5
Olympus Biotech Corporation (Trafermin South) (2010) [62]	1	1		1	1		1	5
Zhang (2019) [63]	1							1
Richard et al (1995) [64]	1			1				2
Fu X et al (2002) [65]	1	1						2
Zheng H‐T et al (2019) [66]	1	1						2
Song Z‐Q et al (2006) [67]	1	1						2
Liu et al (2016) [68]	1	1						2
Xu 2018 [69]	1	1					1	3
Recombinant Human Vascular Endothelial Growth Factor								
Hanft et al (2008) [70]	1	1						2
Platelet Rich Fibrin								
Li (2015) [71]	1	1					1	3
Elsaid (2019) [72]	1	1						2
Tsai et al (2019) [73]	1	1		1	1			4
Ahmed et al (2017) [74]	1	1						2
Driver (2006) [75]	1	1		1	1		1	5
Saldalamacchia (2004) [76]	1	1		1	1		1	5
Kakagia (2007) [77]	1	1		1	1			4
EGF								
Tsang et al. (2003) [78]	1	1		1	1	1		3
Afshari et al (2005) [79]	1	1		1	1			4
Viswanathan et al. (2019) [80]	1	1					1	3
Gomez-Villa et al. (2014) [81]	1	1		1	1		1	5
Singla et al. (2014) [82]	1	1						2
Fernandez-Montequin et al. (2009) [83]	1	1		1	1		1	5

Patient and Public Involvement

No patients or members of the public were directly involved in this research study.

Results

A total of 41 studies were included in this review. Details of the included studies are outlined in Figure 1.

Standard of Care (SOC)

SOC is defined as the standard wound dressing for diabetic foot ulcers that is currently being used. They differ slightly in different hospitals but all of them generally involve wound off-loading, debridement, moist wound care, and alginate wound dressing.

Platelet-Derived Growth Factors/Platelet-Derived Wound Healing Formula

There are a total of six RCTs on the use of platelet-derived growth factors in DFUs [44-49]. All trials compared the use of becaplermin to other adjuncts such as the OASIS wound matrix (Smith & Nephew, London, United Kingdom), a natural extracellular matrix, and TheraGauze (Soluble Systems LLC, Newport, VA), an antimicrobial gauze (n=2), or itself at different concentrations/placebos (n=3).

A double-blinded RCT study involving 382 participants conducted by Wieman et al. (1998) was conducted. It concluded that becaplermin gel 100 micrograms/g significantly increased the incidence of complete wound closure by 43% (p = 0.007) and significantly reduced the time to complete closure of chronic diabetic neuropathic ulcers by 32% (p = 0.013). The safety profile of becaplermin gel was similar to that of placebo gel. Adverse events, such as osteomyelitis and cellulitis, reported during treatment or during a three-month follow-up period were similar in incidence across all treatment groups [44].

The open-label RCT study involving 922 participants conducted by Steed et al. (2006) concluded that PDGF at 100 mug/g had a significant increase in complete healing compared with patients given a placebo. Patients treated with 100 mug/g PDGF with ulcers of baseline area <10 cm had a significant increase in complete healing compared with the placebo (p < 0.007). PDGF also decreased the time to complete healing by 30% (14 weeks versus 20 weeks, p = 0.01). Adverse events were similar in both treatment groups, as were recurrent ulcer rates [45]. See Table 3.

**Table 3:** Summary of studies on the platelet-derived wound healing formula

Source	Participant characteristics	Participant count	Intervention	Control/Comparator	Duration of intervention and follow-up	Outcomes	Significant findings
Niezgoda et al (2005)[46]	At least 1, 1 month non-healing full-thickness diabetic foot ulcer >=18 y/o	73	OASIS wound matrix, Weekly dressing and debridement if necessary	Regranex Gel (becaplermin)	12 weeks; 12 weeks	Incidence of healing in each group at 12 weeks.	At 12 weeks, incidence of complete wound closure of OASIS-treatment was similar to treatment with regranex [p=0.055]
Wieman et al (1998)[44]	Patients with type 1 or type 2 diabetes and chronic ulcers of at least 8 weeks' duration	382	Becaplermin gel 30 mcg/g, 100 mcg/g, saline gauze dressings changed twice daily; medication applied at evening dressing change	Placebo gel	The standardized regimen of good wound care until complete wound closure was achieved or for a maximum of 20 weeks.	Incidence of complete wound closure; Time to complete wound closure	At 20 weeks, the incidence of complete wound closure was higher in 100mcg/g becaplermin gel than placebo gel [50% vs 35%, p=0.007]
							At 20 weeks, the time taken to achieve complete wound closure is shorter in 100mcg/g becaplermin gel by 32% compared to placebo gel [86 vs. 127 days; estimated 35th percentile, p=0.013]
Landsman (2010)[47]	Patients with type 1 or type 2 diabetes and chronic ulcers of at least 8 weeks' duration	TheraGauze + Becaplermin	TheraGauze + Becaplermin	TheraGauze	20 weeks	Rate of wound closure. Wounds achieving closure at 12 and 20 weeks	At 20 weeks, wound closure rates in patients treated with becaplermin were similar compared to those without [p = 0.34]
							At 12 and 20 weeks, the rate of wound closure of both interventions was higher than historical saline-soaked gauze treatment data [Week 12: 46.2% in both groups; Week 20: 61.5% with TheraGauze vs 69.2% with TheraGauze + becaplermin; Week 20: 0.24 cm2/week vs 0.18 cm2/week]
Ma et al (2015)[48]	Type 1 or type 2 diabetes Chronic ulcers of at least 8 weeks' duration	46	Regranex + offloading with a short leg walking cast. Medication applied daily and casts changed approximately every 14 days.	Placebo offloading with a short leg walking cast.	Treatment up to 4 months	Healing rate	At 4 months, the incidence of healing in offloading with a short leg walking cast showed no significant improvement of healing.
Steed et al (2006)[45]	Full-thickness diabetic neurotrophic foot ulcers present for longer than 8 weeks	922	PDGF at 100 mug/g applied once daily	Placebo gel	20 weeks	Complete healing incidence at 20 weeks. Time to complete healing	At 20 weeks, the incidence of complete wound healing for PDGF was higher than placebo gel [50% vs 36%, p < 0.007].
							At 20 weeks, the time to complete healing for PDGF was shorter than placebo gel by 30% [14 weeks vs 20 weeks, p = 0.01]
Melba S et al (2016)[49]	Type 2 diabetes with grade 1 or 2 ulcer, speaks local Kannada and Konkani	50	Foot care education + rhPDGF	rhPDGF or Betadine gel (SOC)	30 days	Time to complete healing of the wound	The combined efficacy of foot care education and rhPDGF resulted in complete closure of the wound with a mean time of 15.91 days compared to the medication intervention (rhPDGF) and the CG in foot ulcers.

Human Umbilical Cord

There was a total of seven randomized controlled trials on cryopreserved human umbilical cord and amniotic membrane [50-56]. A variety of topical compounds were used; in general, they were either dehydrated human amnion and chorion membrane (dHACA) or hypothermically stored amniotic membrane (HSAM).

DiDomenico et al. (2016) reported an interim analysis of a randomized control trial of weekly dHACA applications and SOC against SOC alone. At 12 weeks, 85% of dHACA and SOC-treated DFUs resulted in wound closure compared to 25% in the SOC group. DiDomenico et al. (2018) published the full clinical trial results three years later with a larger sample size of n=80 [53]. The results were consistent with the interim results and showed that healing of DFU with dHACA and SOC was 85% at 12 weeks compared to 33% using SOC alone (p = 6.0 × 10−6). Subsequently, Glat et al. (2019) conducted a head-to-head trial comparing SOC and dHACA against SOC and Apligraf, a well-established tissue-engineered skin substitute, for the treatment of DFUs. At 12 weeks, 90% of wounds had closed for the SOC and dHACA group compared to 40% for the Apligraf and SOC group (p=4.9 × 10-5) [50].

Serena et al. (2020) identified another different form of amniotic membrane, HSAM, which involves hypothermal storage. Clinical benefits are due to its intact amniotic membrane, and refrigerator storage maintains membrane cell viability. The efficacy of HSAM in healing DFUs was studied. There were 76 patients in this trial (HSAM and SOC group (debridement, infection elimination, use of dressings, and offloading by total contact casting), n=38; SOC alone group n=38). At 12 weeks, wound closure for HSAM and SOC was significantly greater than SOC alone with 60% compared to 38%, respectively (p=0.04) [51].

Subsequently, Glat et al. (2019) conducted a head-to-head trial comparing dHACA+SOC with TESS/Apligraf (Organogenesis, Canton, MA) + SOC, a well-established tissue-engineered skin substitute for the treatment of DFUs. At 12 weeks, 90% of the wounds had closed for the dHACA+SOC group compared to 40% for the TESS group [50]. See Table 4.

**Table 4:** Summary of studies on the human umbilical cord dHACA: dehydrated human amnion and chorion allograft; DFU: diabetic foot ulcer

Source	Participant characteristics	Participant number	Intervention	Control/Comparator	Duration of intervention and follow-up	Measures of effect	Significant findings
Serena TE et al (2020) [51]	All included subjects presented with a DFU located below the medial aspect of the malleolus extending at least through the epidermis into the dermis, subcutaneous tissue, muscle, or tendon but not into bone	76	HSAM = (Hypothermically stored amniotic membrane)	SOC = debridement, infection elimination, use of dressings, and offloading by total contact casting (TCC)	12-week treatment phase and a 4-week follow-up phase. Total 16 weeks	Frequency of wound closure, Time to wound closure, The number of subjects showing >60% reduction in baseline ulcer area, The number of subjects showing >60% reduction in baseline ulcer depth, The number of subjects showing >75% reduction in baseline ulcer volume.	At 12 and 16 weeks, wound closure for HSAM was significantly greater than SOC (p = 0.04) [Week 12: 60 vs 38%; Week 16: 63 vs 38%]
							Probability of wound closure increased by 75% [Hazard Ratio = 1.75; (95% CI: 1.16-2.70)]
							HSAM showed >60% reductions in area (82 vs 58%; p = 0.02) and depth (65 vs 39%; p = 0.04) versus SOC
							The K–M median time to wound closure for HSAM-treated ulcers was 11 weeks. For SOC-treated ulcers, the K–M median time to wound closure was not attained by 16 weeks
DiDomenico LA et al (2018)[53]	Patient's wound diabetic in origin and larger than 1 cm2, Wound present for a minimum of 4-week duration, with documented failure of prior treatment to heal the wound	80	Weekly application of dHACA+SOC (dehydrated human amnion and chorion allograft)	off-loading, appropriate debridement, and moist wound care (daily)	12 weeks, 12 weeks	Mean time to wound healing within 12 weeks, Number of subjects with a healed wound at 12 weeks	At 12 weeks, dHACA heals DFUs significantly greater than SOC [(85% (34/40) vs 33% (13/40)]
							At 12 weeks, the mean time to heal was significantly shorter for dHACA compared to SOC (P = .000006) [37 days vs 67 days]
Glat P et al (2019)[50]	Index wound is ≥1 and <25 cm2 Index wound present for a minimum of 4 wk duration and a maximum of 1 y	60	dHACA+SOC (non-adherent dressing (Adaptic Touch; Acelity), steri-strips + moisture-retentive dressing (hydrogel bolster) + padded 3-layer dressing Dynaflex (Acelity)	TESS/ Apligraf+SOC	12 weeks, 12 weeks	Mean time to heal within 6 weeks and 12 weeks, Proportion of wounds healed at study completion (12 weeks)	At 6 and 12 weeks, the mean time to heal for dHACA was higher than TESS [Week 6: 24 days (95% CI, 18.9–29.2) versus 39 days (95% CI, 36.4–41.9); 12 weeks: 32 days (95% CI, 22.3–41.0) vs 63 days (95% CI, 54.1-72.60]
							At 12 weeks, dHACA had a higher proportion of wounds healed than TESS [90% (27/30) vs 40% (12/30)]
							dHACA heals diabetic foot wounds more reliably, statistically significantly faster
Zelen, C. M. et al (2015)[52]	Index wound is ≥1 and <25 cm2, Ulcer duration of ≥6 weeks, unresponsive to standard wound care	60	Epifix (dHACA)	Apligraft or collagen-alginate dressing (SOC)	6 weeks	Percent change in complete wound healing and proportion of patients with complete wound healing after 4 and 6 weeks, Percent change in wound area per week, Median time to wound healing	The proportion of patients in the EpiFix group achieving complete wound closure within 4 and 6 weeks was 85% and 95%, significantly higher (P ≤ 0·003) than for patients receiving Apligraf (35% and 45%), or standard care (30% and 35%).
							After 1 week, wounds treated with EpiFix had reduced in area by 83·5% compared with 53·1% for wounds treated with Apligraf.
							Median time to healing was significantly faster (all adjusted P‐values ≤0·001) with EpiFix (13 days) compared to Apligraf (49 days) or standard care (49 days).
Tettelbach, W. et al (2019) [54]	HgA1c	98	Dehydrated human amnion/chorion membrane allograft (dHACM)	Standard of care with alginate wound dressing	12 weeks	Percentage of study ulcers completely healed in 12 weeks	Participants receiving weekly dHACM significantly more likely to completely heal than those not receiving dHACM (ITT-70% versus 50%, P = 0.0338, per-protocol-81% versus 55%, P = 0.0093).
							Cox regression analysis showed that dHACM-treated subjects were more than twice as likely to heal completely within 12 weeks than no-dHACM subjects (HR: 2.15, 95% confidence interval 1.30-3.57, P = 0.003)
Snyder, R. J. et al (2016)[56]	Type 1 or 2 diabetes, HbA1c <12%, At least 1 wound of ≥1 and <25 cm2; at least Wagner grade 1	29	Dehydrated amniotic membrane allograft (DAMA)	Debridement, hemostasis, moist wound dressings, offloading where appropriate with a DH Walker boot (SOC)	6 weeks	Proportion of subjects with complete wound closure (complete reepithelization)	33% of subjects in the DAMA+SOC cohort achieved complete wound closure at or before week 6, compared with 0% of the SOC alone cohort (intent-to-treat population, P = 0.017)
Tettelbach, W. et al (2019)[55]	Subject has completed 14‐d run‐in period with ≤30% wound area reduction post‐debridement, Area post‐debridement of 1 to 15 cm2, Present for ≥30 d	134	Dehydrated human umbilical cord allograft (EpiCord)	Alginate wound dressings (SOC)	12 weeks	Percentage of complete closure; healing rate of the study ulcer within 12 weeks	ITT analysis showed that DFUs treated with EpiCord were more likely to heal within 12 weeks than those receiving alginate dressings, 71 of 101 (70%) vs 26 of 54 (48%) for EpiCord and alginate dressings, respectively, P = 0.0089.
							Healing rates at 12 weeks for subjects treated PP were 70 of 86 (81%) for EpiCord-treated and 26 of 48 (54%) for alginate-treated DFUs, P = 0.0013.

FGF

Uchi et al. (2009) investigated the clinical efficacy of different doses of bFGF administered via a topical spray. The area of ulcer decreased by 75% or more in 57.5%, 72.3%, and 82.2% in the placebo, 0.001% bFGF, and 0.01% bFGF groups, respectively. Differences were significant between the 0.01% bFGF and placebo groups (p = 0.025). Furthermore, dose-dependent, linear increases were noted using the Cochran-Armitage test (p =0.009). The cure rate was 46.8%, 57.4%, and 66.7%, respectively. This result, however, did not have statistical significance. The reduction in ulcer depth was also not statistically significant. He also concluded that recommended treatment length should only be up to eight weeks. His results showed bFGF had wound healing accelerating effects [57].

Morimoto et al. (2013) investigated a novel therapy involving bFGF and artificial dermis, which has been reported to accelerate dermis-like tissue formation. This dermis, known as collagen/gelatin sponge (CGS), was recently developed and can sustain the release of bFGF for over 10 days. Similar to Uchi et al. (2009), there was no significant difference between the two doses for wound bed improvement and the percentage of wound reduction over two weeks. This suggests that there is no requirement for such a large concentration of bFGF for effective healing to take place. However, there was a significant difference in the percentage of wound bed improvement between the two doses (p=0.04). Finally, all outcomes were significant when the doses were compared to the null hypothesis of 10% healing within two weeks [58]. The combined therapy with CGS and bFGF could be a promising treatment that is comparable to skin substitutes containing living cells in terms of cost and usability.

Combination therapy has also been investigated. Steed et al. (1992) assessed the efficacy of topically applied activated platelet supernatant (CT-102 APST) vs placebo in treating DFU [59]. CT-102 APST contains multiple growth factors, including aFGF and bFGF. In the CT-102 group, 5/7 ulcers were healed by week 15, but only 1/6 ulcers were healed by week 20 in the placebo group. It was concluded that CT-102 significantly accelerated wound closure in diabetic leg ulcers when administered as part of a comprehensive program for the healing of chronic ulcers. Santoro et al. (2018) investigated concentration growth factors (CGFs) for the treatment of non-healing vascular ulcers. These factors included FGF, PDGF, and VEGF, and play a role in cell proliferation, vascular maintenance, and angiogenesis. The topical application of CGF was compared to standard dressing weekly for six weeks. After six weeks, there was a reduction of >50% of the area in lesions in 61.3% in the CGF group compared to 6.7% in the standard dressing group. Out of the 30, 6/9 of them were patients with arterial diabetic ulcers who achieved this endpoint [60]. Both these studies have concluded the effectiveness of combined therapy compared to standard dressing.

The largest clinical trial was recently conducted to investigate the effect of trafermin in DFU healing. It involved a total of 368 patients over two regions (Northern and Southern Europe) where 0.01% of trafermin spray was compared to a matching placebo spray. In the Northern study, 21.0% of patients treated with trafermin achieved complete wound healing after 12 weeks compared to 16.7% of patients treated with placebo. Fifty-three point five percent (53.5%) of patients treated with trafermin achieved wound regression with 40% or more in six weeks compared to 55.9% of patients treated with placebo. In the Southern study, the wound closure rate of DFU in 12 weeks is 14.1% in trafermin-treated patients compared to 10.8% in placebo-treated patients. Relative wound area regression of 40% or more at six weeks is 60.9% in trafermin-treated patients vs 52.9% in placebo-treated patients [61-62]. See Table 5.

**Table 5:** Summary of studies on fibroblast growth factor

Source	Participant characteristics	Participant number	Intervention	Control/Comparator	Duration of intervention and follow-up	Measures of effect	Significant findings
Zhang (2019)[63]	Diabetes mellitus patients complicated with deep second-degree burn	80	basic fibroblast growth factor (bFGF)	polymyxin B ointment	28 days	Time of wound pain, wound scarring, time to wound healing, Levels of AGEs and VEGF	Time to wound healing, the pain relief in the intervention was significantly shorter than that in the control group, 24.1 days in the intervention vs 31.9 in the control (p<0.05).
Uchi et al (2009) [57]	Stage II Wagner (900 mm2 or less), ABI > 0.9 if no DP/PT pulse	150	5 puffs once a day, 0.001% bFGF, 0.01% bFGF	placebo	total duration 8 weeks	Percentage of patients showing ≥75% reductions in ulcer cure rate	Wound healing accelerating effects noted for bFGF on diabetic ulcers.
							A significant difference in the percentage of patients showing ≥75% ulcer reduction in the interventional groups (p = 0.025).
							Cure rate was 46.8% (22/47), 57.4% (27/47), and 66.7% (30/45) in the placebo, 0.001% bFGF and 0.01% bFGF groups, respectively. Insignificant
Richard et al (1995) [64]	Grade I-III Wagner, the largest part of the ulcer must be more than 0.5 cm, VPT >30 V, No significant peripheral vascular disease or wound infection, Tight glycemic control	17	Local application of bFGF	Placebo	1st 6 weeks, once a day, Last 2 weeks, Twice a week	Cure rate, Weekly reduction in ulcer perimeter and area, Percentage of healed area	Weekly reduction in ulcer perimeter and area was identical in both groups
							No significant difference between the rate of linear advance of healing (P = 0.08)
							Three of nine ulcers healed compared with five of eight in the placebo group (NS)
							Topical application of bFGF has no advantage over placebo for healing chronic neuropathic diabetic ulcers of the foot
Morimoto N et al (2013) [58]	20 years or older, not healing for at least 4 weeks with conventional treatments; If chronic ulcer present, SPP≥ 30 30 mmHg, Controlled diabetes	14	CGS (artificial dermis, collagen/gelatin sponge), capable of sustained high-dose bFGF release for over 10 days, Treated with CGS impregnated with bFGF at 7 or 14 μg/cm(2) after debridement	Low-dose bFGF	CGS application for 14 days, with follow-up until 28 days	Wound bed improvement, Percentage of wound bed improvement, Percentage of wound reduction, granulation area	16/17 patients showed wound bed improvement, significantly superior to the null hypothesis of 10% (p< 0.001)
							No significant difference between the low-dose group and high-dose group (p=1.00)
							A first-in-man clinical trial of CGS showed the safety and efficacy of CGS impregnated with bFGF in the treatment of chronic skin ulcers. This combination therapy could be a promising therapy for chronic skin ulcers.
Fu X et al (2002) [65]	-	185 (diabetes)	rbFGF				173/185 chronic dermal ulcers treated with rbFGF healed within 6 weeks
Steed DL et al (1992) [59]	Non-healing ulcer of > 8 wk duration peri-wound transcutaneous oxygen tension > 30 mmHg, platelet count > 100,000/mm3, no wound infection.	13	CT-102 APST (PDGF, PDAF, EGF, PF-4, TGF-beta, aFGF, and bFGF)	Placebo (normal saline)	20 weeks of treatment	Cure rate (100% epithelization), Percent reduction in ulcer area, Reduction in ulcer volume, ulcer area	5/7 ulcers were healed by 15 wk, but only 1/6 ulcers was healed by 20 wk with a placebo (P < 0.05).
							The average percent reduction in ulcer area at 20 wk was 94% for CT-102 vs. 73% for placebo.
							Significant daily reduction in ulcer volume and area for CT-102 vs placebo (P < 0.05 for both)
							CT-102 significantly accelerated wound closure in diabetic leg ulcers when administered as part of a comprehensive program for the healing of chronic ulcers.
Zheng H‐T et al (2019) [66]	-	32	bFGF group (hypoglycemic + anti‐infective drugs + rb‐bFGF	Control group (hypoglycemic + anti‐infective drugs	-	Healing rate, healing time	Healing rate of bFGF group (94%) was significantly higher than control group (62%) (χ2 = 4.96, P < 0.05).
							Healing time of the bFGF group (29.34 ± 46) was significantly shorter than control (38.23 ± 2.87) (29.34days] (t = 11.06, P < 0.05).
Song Z‐Q et al (2006) [67]	-	29	rhEGF combined bFGF, rhEGF, bFGF, RhEGF, and bFGF were sprayed over the wound by 1 500 IU per time and 720 AU per time	Saline	-	-	On the 3rd day and 7th day after treatment, the growth was similar in every group (P > 0.05).
							On the 14th day, the granulation tissues of the E+F group grew better than that of the E group, F group, and the saline group (P < 0.05).
							There is a cooperation effect of rhEGF combined with bFGF in diabetic wound therapy.
Olympus Biotech Corporation (2010) (Trafermin North) [61]		188	Trafermin 0.01% spray 5 puffs when ulcer <6cm, 10 puffs when ulcer >6cm microgram) sprayed onto each half of the wound surface	Matching placebo spray	Treatment: 12 weeks Follow-up: 9 months	Wound closure rate of ulcer after 12 weeks, Wound area regression of >40% at 6 weeks	21.0% of patients treated with trafermin with complete wound healing after 12 weeks compared to 16.7% of patients treated with placebo.
							53.5% of patients treated with trafermin with >40% or more in 6 weeks compared to 55.9% of patients treated with placebo.
							Serious adverse events in 19.05% of trafermin-treated patients compared to 25.49% of patients treated with placebo.
Olympus Biotech Corporation (2010) (Trafermin South) [62]		180		Matching placebo spray			The wound closure rate of DFU in 12 weeks is 14.1% in trafermin-treated patients while the rate is 10.8% in placebo-treated patients.
							Relative wound area regression of 40% or more at 6 weeks is 60.9% in trafermin-treated patients vs 52.9% in placebo-treated patients.
							23.3% of trafermin patients with serious adverse events compared to 16.67% of placebo-treated patients.
Liu et al (2016)[68]	Diabetic foot ulcers (Wagner grade 2‐3)	60	Autologous APG group	Rb-bfgf gel group	Treatment: 8 weeks	Healing rate Healing time	After 8 weeks, in the APG treatment group and control group, the healing rate of overall sample ulcer (P=0.005), sinus ulcer (P=0.033), Wagner 3 (P=0.030) differed significantly but did not significantly differ in superficial ulcer (P=0.106) or Wagner 2 ( P=0.106).
							The autologous platelet-rich gel can effectively increase the curative rate of diabetic feet and shorten healing time.
							APG vs bfgf (overall ulcer, superficial ulcer, sinus ulcer, Wagner 2 and Wagner 3).
							Ulcer healing time was 31 d vs 41.5 d, 23 d vs. 32 d, 32 d vs.56 d, 25 d vs. 32 d, 38 d vs. 56 d, with a significant difference between the two groups (P<0.05).
Santoro et al (2018) [60]	-	61	Concentrated growth factors (CGFs) rich in platelet‐derived growth factors (PDGF, TGF‐beta1, TGFbeta2, FGF, VEGF, IGF) and CD34+ stem cells, topical application of CGF weekly	Standard dressing (application of polyurethane film or foam weekly)	Treatment: 6 weeks	Reduction of ≥ 50% surface and volume of lesions	At 6 weeks, a reduction of ≥50% of surface and volume of lesions for CGF was significantly greater than control (p <0.001) (19 of 31 (61.3%) vs 2 of 30 (6.7%)).
							CGF therapy was more effective than standard dressing for the treatment of non‐healing ulcers of multiple etiologies.
Xu (2018) [69]	-	199	rh-EGF, aFGF, rh-EGF, and aFGF (n=50 each), Daily dressing change; growth factor reagents applied topically when dressing	normal saline control group (n=49)	-	Rate of wound healing (Epidermal healing rate and granulation tissue growth)	At 4 days, no significant difference between all groups.
							>4 days, wound healing for rh-EGF+aFGF had a marked positive effect compared with control
							Time to complete wound healing 41.83 days in aFGF vs 47.52 days with saline (p-value insignificant)
							When aFGF combined with EGF, then 36.31 v 47.52 and significant

VEGF

The VEGF trial of significance included 29 patients that received topically applied Telbermin (72 µg/cm2) and 26 patients that received placebo (n=26) treatment [70]. Telbermin showed more complete ulcer healing (p =0.39) (41.4% vs 26.9% at day 43). Telbermin showed a faster time to complete ulcer healing (25th percentile of 32.5 days vs 43.0 days). Topical application of Telbermin 72 microgram/cm^2^ three times a week for up to six weeks appears to be well-tolerated [70].

PRF

The largest clinical trial was published by Li in 2015, which included 117 patients who received topical autologous platelet gel (APG) application on the wound beds before Suile™ baseline administration. This trial was also the trial with the fastest reported time to complete healing. The APG application was compared against the control of Suile™ wound dressing. The Kaplan-Meier time-to-healing from the intention to treat (ITT) population was significantly different between the two groups. Furthermore, faster healing velocity in the APG group than in the control group (p = 0.020). After the 48^th^ day, there were similar maximum median reduction rates of 100% [71].

The most recent trial conducted using APG was by Elsaid in 2020 with 24 patients, where vaseline and platelet-rich plasma (PRP) gel was used as the intervention, compared with a control of daily dressing with normal saline [72]. The percentage reduction in the longitudinal and horizontal dimensions of the DFU was significantly greater in the intervention than in the control group (43.2% vs 4.1%) and (42.3% vs 8.2%), respectively. The time required to maximum healing was significantly shorter in the intervention than in the control group (p = 0.0001) (6.3 ± 2.1 vs 10.4 ± 1.7 weeks).

Tsai 2019 was the only trial to use a novel injection method as a mode of administration of PRP as compared to the other trials where it was administered in gel form. This trial compared PRP injection to placebo of traditional collagen-based foam dressings. At four weeks, wound size reduction to <25% of the original area was noted with PRP. The healing process of PRP was statistically significant (p <0.0001). Within the last three weeks, >90% of the subjects had wounds of <10% of their original size compared to the placebo group. In the placebo group, the wound area remained at 25-50% of the original size at the end of the trial [73]. See Table 6.

**Table 6:** Summary of studies on platelet-rich fibrin

Source	Participant characteristics	Participant number	Intervention	Control/Comparator	Duration of intervention and follow-up	Outcomes	Significant findings
Tsai et al (2019)[73]		17 with diabetes, 11 without	Platelet-derived patch treatment and PRP injection	Placebo (traditional, silver-impregnated, collagen-based foam dressings)	12 weeks	Change in wound size, time to healing	At 4 weeks, wound size reduction to <25% of the original area was noted with PRF. The healing process of PRP was statistically significant (P<0.0001)
							Within the last 3 weeks, >90% of the subjects had wounds of <10% of their original size in the last three weeks of the trial compared to the placebo group, where wound area remained at 25-50% of original size at end of the trial.
Ahmed et al (2017) [74]	Both sex from 18 to 80 years, nonhealing for >6 weeks, Grade I-II	56	Autologous platelet gel	Antiseptic ointment dressing	12 weeks	Wound closure	At 12 weeks, significantly greater healing rate in the PRP group (86% vs 68%)
							Rate of healing per week greater during the first 8 weeks; declines afterward.
							The use of platelet gel showed a lower rate of wound infection.
Elsaid (2020)[72]	Non-infected chronic foot ulcer confined to one anatomical site. Chronicity was defined as a non-healing ulcer for 12 or more weeks, Patients with chronic limb ischemia, osteomyelitis, or exposed tendons, ligaments, or bones at the base of the ulcer were excluded	24	Vaseline and PRP dressing daily	Daily dressing with normal saline	20 weeks	Time to healing, Percentage with complete healing, Percent reduction in the longitudinal and horizontal dimensions	By the end of the trial, 3 (25%) patients in the intervention achieved complete healing vs none of the control.
							8.3% of participants receiving the intervention and 41.6% of control patients did not show any response to treatment.
							Percent reduction in the longitudinal and horizontal dimensions of the DFU was significantly greater in the intervention than in the control group (43.2% vs 4.1%) and (42.3% vs 8.2%), respectively.
							The time required to maximum healing was significantly shorter in the intervention than in the control group (P= 0.0001) ((6.3 ± 2.1 vs 10.4 ± 1.7 weeks).
Li (2015) [71]	An ulcer that did not improve significantly after at least 2-week ulcer standard treatments; the 2-3 Wagner's grade for the DFUs	117	Topical APG application on the wound beds before a Suile baseline administration.	Covered with Suile wound dressing, which contained vaseline mostly and was occlusive	12 weeks	Time to healing, Rate of survival, and recurrence within follow-up	Kaplan-Meier time-to-healing from the ITT population was significantly different between the two groups [(36 (IQR 30–84) days for the APG group, 45 (IQR 18–60) days for the control group)].
							Faster healing velocity in the APG group than in the control group (p = 0.020). After the 48th day, similar maximum median reduction rates of 100%.
Driver (2006)[75]	Ulcer of at least 4 weeks’ duration wound area. Non-infected and without exposure of the bone, muscle, ligaments, or tendons	72	PRP gel	A normal saline gel was applied following wound bed preparation			By the end of the trial, 13 of 19 (68.4%) patients in PRP gel and nine out of 21 (42.9%) patients in the control group healed (P = 0.125, two-sided Fisher’s exact test).
							High percent proportion of completely healed wounds in PRP gel versus control groups (95% CI: 47.5-89.3% vs 21.7-64.0%).
Saldalamacchia (2004) [76]	Wagner Grade II/III ulcers, lasting for at least 8 weeks and with no signs of infection at recruitment.	14	Platelet gel	SOC			At five weeks, significantly larger average reduction rate in patients treated with platelet gel.
							100% improvement in PG wounds: two ulcers healed completely versus one in the ST group; five with a significant reduction in wound area versus 5 unchanged and 1 worsening in the ST group.
							A higher proportion of complete healing of reduction of 50% more in the platelet gel group (71% vs 29%; OR 6.2; 95% CI 0.6-63)
Kakagia (2007)[77]	Ulcers of at least 3 months post-debridement	54	B: Autologous growth factors delivered by Gravitational Platelet Separation System and covered by a vapor-permeable film	A: ORC/collagen biomaterial and covered by a vapor-permeable film, C: Combination of both by means of covering the plasma-centrifuged concentrate that was produced by the GPS and applied at the ulcer bed with the ORC/collagen biomaterial and covered by a vapor-permeable film			Significantly greater reduction of all three dimensions of the ulcers in Group C compared to Groups A and B (P<0.001).
							No significant reduction in ulcer dimensions in group B versus A.
Shailendra (2018) [84]		55	Platelet-rich plasma (PRP)	SOC	28 days	Time to wound healing	Complete healing occurred in all patients in the study group in (mean score and standard deviation), 36.7±3 days compared with 60.6±3.7 days in the control group (p<0.0001).

EGF

There was a total of six randomized controlled trials on EGF, where two different topical compounds were used; three trials using recombinant EGF and three trials using EGF [78-83].

Tsang et al. (2003) conducted the first randomized clinical trial. It demonstrated that the application of topical Actovegin cream with 0.04% (wt/wt) human epidermal growth factor (hEGF) caused more ulcers to heal by 12 weeks. Twenty out of 21 patients in the 0.04% (wt/wt) hEGF group achieved complete healing. Healing rates were 42.10% and 95% for the control and the 0.04% (wt/wt) hEGF groups, respectively. hEGF has been shown to increase the rate of healing compared with the other treatments (p = 0.0003) [78].

The most recent clinical trial was conducted by Viswanathan et al (2019), where a recombinant hEGF gel-based product (Regen-D) was compared to an alternative placebo group. The healing time of the wound among the Regen-D subjects was significantly less than the placebo group (45 ± 12 vs 72 ± 18 days, p < .0001), and even showed a better blood glucose level. After the completion of the study period of 30 days, 78% of subjects receiving Regen-D attained complete healing of ulcers compared with 52% of subjects receiving placebo [80]. See Table 7.

**Table 7:** Summary of studies on human epidermal growth factor

Source	Participant characteristics	Participant number	Intervention	Control/Comparator	Duration of intervention and follow-up	Outcomes	Significant findings
Tsang et al. (2003) [78]		61	Actovegin 5% cream and 0.02% hEGF, Actovegin 5% cream, and 0.04% hEGF	Actovegin 5% cream	12 weeks	Proportion with complete wound healing, Wound healing rate	20 of 21 patients (95.3%) in the 0.04% (wt/wt) hEGF group achieved complete healing
							Healing rates were 42.10 and 95% for the control and the 0.04% (wt/wt) hEGF groups, respectively
							Median time to complete healing in the 0.04% (wt/wt) hEGF group was 6 ± 1 weeks (CI 4.22–7.78) (log-rank test, P = 0.0003)
Afshari et al. (2015) [79]		50	1 mg of recombinant human EGF/1000 mg of 1% silver sulfadiazine in a hydrophilic base	Placebo	4 weeks	The proportion of complete wound healing	Average wound closure in the treatment group was significantly greater than in the placebo (71.2 vs. 48.9%, p = 0.03)
							Amongst those with grade I ulcers, 15 (50%) of patients receiving EGF had >70% closure compared to 3 (15%) on placebo (p=0.05)
							Amongst those with grade II ulcers, 7 (23.3%) of patients receiving EGF had complete closure compared to 2 (10%) on placebo (p=0.3)
Viswanathan et al. (2019)[80]		50	hEGF daily	Placebo	30 days	The proportion with complete wound healing, Time to complete wound healing	Complete healing of ulcers was observed in 21 (78%) subjects in group 1, whereas only 12 (52%) subjects in group 2 reported of complete healing of ulcers after completion of the study period of 30 days.
							Shorter time taken to heal in patients receiving EGF than placebo (Mean in days ±SD: 45 ± 12 vs 72 ± 18;
Gomez-Villa et al. (2014) [81]		31	Thrice-per-week intralesional application of rhEGF (75 μg) (n=15)	Placebo (n=16)	8 weeks		Compared to the placebo group, more ulcers achieved complete healing in the rhEGF group (rhEGF, n = 4; placebo, n = 0; p = 0.033); ulcers in the rhEGF group decreased in area size (12.5 cm2 [rhEGF] vs. 5.2 cm2 [placebo]; p = 0.049)
Singla et al. (2014) [82]		50	Topical rhEGF (n=25)	Betadine dressing (n=25)	8 weeks	The proportion with complete wound healing	12 patients receiving rhEGF vs 3 with betadine achieved complete wound healing
Fernandez-Montequin et al. (2009) [83]		149	EGF 75 µg three times per week (n=53), EGF 25 µg three times per week (n=48)	Placebo 3 times a week (n=48)	8 weeks	Granulation tissue covering ≥ 50% of the ulcer at 2 weeks, end‐of‐treatment complete granulation response, time-to-complete response	Granulation tissue covering ≥ 50% of the ulcer at 2 weeks was achieved by 19/48 controls versus 44/53 in the 75 µg group [odds ratio (OR): 7·5; 95% confidence interval (CI): 2·9–18·9] and 34/48 in the 25 µg group (OR: 3·7; 1·6–8·7)
							End‐of‐treatment complete granulation response (28/48 controls, 46/53 with 75 µg, and 34/48 with 25 µg EGF)
							time‐to‐complete response (controls: 5 weeks; both EGF dose groups: 3 weeks)
							Wound closure after follow‐up (25/48 controls, 40/53 with 75 µg, and 25/48 with 25 µg EGF) was also treatment-dependent

Discussion

Our literature review seeks to elucidate some of the more promising and effective biologic agents that improve the time to wound healing and percentage of wound recovery. Covering 41 randomized-controlled trials from 1992-2020, there are multiple ongoing trials that seek to further refine the current evidence and translate research into patient benefit. In summary, we included six RCTs on PDGF [44-49], seven on the human umbilical cord or amniotic membrane [50-56], 13 on FGF [57-69], eight on PRP [71-77,84], six on HEGF [78-83], and one on VEGF [70]. Most of these trials demonstrated that biologic adjuncts are efficacious in reducing the time to complete wound healing or increasing the proportion of patients with completely healed ulcers.

HUC has shown the most consistency in its performance across multiple RCTs. The time to wound healing in all seven HUC interventions was significantly shorter than that of a placebo while the percentage of patients with complete ulcer healing was also significantly greater in HUC [50-51,53]. These promising results have been noticed by pharmaceutical companies. Amniox (Miami, FL) has recently announced the initiation of two phase III trials involving the use of cryopreserved umbilical cord TTAX01 allograft to treat complex Wagner grade III-IV diabetic foot ulcers, with the enrolment of 440 patients in total [85]. The study will evaluate the benefits and risks of more complex non-healing DFUs with high-risk factors. These factors include ulcer depth indicating exposed bone, tendon, muscle, and/or joint capsule, and clinical suspicion of osteomyelitis.

It is unclear if FGF is able to significantly improve the time to wound healing or the cure rates in diabetic foot ulcers. There is a discrepancy in results between studies done in different regions. Studies in Japan [57] and Europe [61-62,64] report an insignificant change in cure rate compared to control. However, it should be noted that the study by Richard et al. may be unreliable due to its small sample size. Conversely, studies on FGF in China seem to have a markedly improved cure rate and reduced time to wound healing compared to control [66-69]. Thus, it may be desirable to conduct a cross-regional study to better investigate the efficacy of FGF across regions, as it remains unclear if the results can be attributed to such differences.

VEGF showed a reduced time to complete wound healing from a single trial. However, the rate of ulcer healing was insignificant. It is a promising adjunct, but more studies are required before any conclusion on its efficacy can be properly established. Significance was not reached in the proportion of completely healed wounds or change in healing time. It should be noted, though, that this trial may be underpowered to detect the impact of VEGF on wound healing [70].

PDGF also has had mixed results based on current available RCTs. Of the five RCTs that reported the percentage of patients with complete ulcer healing [44-48], there was no significant improvement in three RCTs [46-48]. However, of the two RCTs that measured time to wound healing, both were able to significantly reduce time to wound healing by an intervention [44-45]. Unfortunately, a recent phase III study conducted by Adocia (France) on diabetic foot ulcer candidate BioChaperone^TM^ was discontinued due to a lack of efficacious results, citing a lack of uniformity in the standard of care in diabetic type wounds. Hence, the efficacy of PDGF is still unclear given the inability to replicate results across large trials [86].

PRF has shown consistency in increased rate and incidence of wound healing/closure. Apart from the trial by Kakagia [77], which showed an insignificant reduction in ulcer dimensions compared to control, all other studies demonstrated significant improvements in one or more of the measured outcomes. These include time to complete ulcer healing, percent of patients with complete ulcer healing, and reduction in ulcer size. At the time that this review was written, there were more published preliminary trials investigating and demonstrating PRF’s efficacy [87-88]. Andreas et al. demonstrated that positive wound healing effects were all observed within the first three to six Vivostat PRF® (Lillerød, Denmark) applications, with a significant increase in the incidence of complete wound healing compared to control [87]. Currently, out of the trials involving PRF in an intervention arm, the largest trial involves 118 patients. Trials that enroll a larger amount of patients may further elicit and quantify the clinical efficacy of PRF.

Uncontrolled diabetes and infected diabetic wounds were exclusion criteria for most trials. However, two studies included these parameters and showed promising results for these patients. Uchi (2009) included patients with uncontrolled diabetes (HbA1c >/ 8.0%) in his investigation, with 21/49 patients receiving 0.001% bFGF with uncontrolled diabetes [57]. Interestingly, stratified analysis conducted with respect to control of blood glucose levels showed that it did not affect the efficacy of bFGF. The drug was significantly more effective in 0.01% bFGF compared to the placebo group, although unspecified in treatment outcome. Marston (2019) conducted an open-label trial (n=32) that involved 20 patients with osteomyelitis [89]. Cryopreserved human umbilical cord (TTAX01) was used in the intervention with 16/32 (50%) of patients achieving wound healing within 16 weeks. Among the patients with osteomyelitis, 60% showed healing in 12.8 weeks while the mean time to healing was 12.8±4 weeks. Following TTAX01 treatment, the overall healing rate was favorable compared to the US Wound Registry standard of care success rates. They reported 45% healing regardless of the time period.

Limitations of this review

The non-standardization in outcome measures results in difficulty in comparing results across studies. There is great diversity in measured outcomes related to wound healing, the variable follow-up periods in each study, and differences in populations studied. This has resulted in significant clinical inconsistency. The various outcome measurements delineating wound healing included wound epithelialization, wound closure, and complete wound healing.

A confounding factor we have identified is diabetic control in the studied subjects. Multiple studies omitted data related to the control of diabetes in patients. While all studies included are randomized controlled trials, it was not possible to ascertain if the subjects’ diabetic control was balanced between all arms of each trial. It is well-studied that better diabetic control would result in significant improvement in wound healing metrics [90]. To establish the comparative value of agents, it would be imperative to take into account the level of diabetic control.

Furthermore, there is heterogeneity in the treatment used under the control group. Out of 41 studies, only 10 studies included the use of placebos. Even amongst the 11 studies that employed standard of care as the control, there is heterogeneity in treatment. For example, Serena TE et al (2020) used dressings and offloading by total contact casting with debridement as necessary [51], compared to Melba S et al. (2016) [49], which employed Betadine gel. As such, when comparing intervention against control for statistical significance, the results may vary depending on the control. Better designed and higher-powered studies would be required before definitive conclusions can be drawn.

Topical biologics are heavily dependent on the healthcare personnel administering the topical. In any type of wound, effective debridement is key to the ability of the growth factor to reach the endothelial cells. Hence, the skill of a healthcare worker also plays a large role in the efficacy of the biologic, and this would be a factor that is difficult to standardize across RCTs.

## Conclusions

At present, targeting the wound healing pathway via the extrinsic administration of growth factors may be a promising option to augment wound healing in diabetic patients. Human umbilical cord treatments seemed to be the most effective, with a consistent incidence of complete ulcer healing and reduced time to complete healing. FGF only showed slight benefits, with few studies showing no statistical difference from placebo. Certain agents, such as VEGF and PRF, demonstrated efficacy in individual trials, but a definite conclusion is yet to be drawn due to small sample sizes. Growth factors have also shown promise in specific subgroups at risk of significantly impaired wound healing, such as those with a history of secondary infection, and future studies may investigate this further. As diabetes mellitus impairs multiple stages of wound healing, combining growth factors may prove to be an area of interest. Evidence from this systematic literature review suggests that topical adjuncts probably reduce time to wound closure, reduce healing time, and increase the healing rate in patients with chronic DFUs.
